# The Monomer Containing Cyano-Oxazine-Trifluoromethyl Groups for Enhancing Epoxy Resin: Thermal Stability, Flame Resistance and Mechanical Behaviors

**DOI:** 10.3390/ma18184279

**Published:** 2025-09-12

**Authors:** Cong Peng, Yuhang Liu, Duo Chen, Zhanjun Wu

**Affiliations:** 1 College of Fiber Engineering and Equipment Technology, Jiangnan University, Wuxi 214122, China; pc@jiangnan.edu.cn (C.P.); 6233017073@stu.jiangnan.edu.cn (Y.L.); 2 School of Material Science and Engineering, Zhengzhou University of Aeronautics, Zhengzhou 450046, China; chenduo@zua.edu.cn

**Keywords:** epoxy resin, benzoxazine, phthalonitrile, flame retardancy, thermal stability

## Abstract

To impart high flame resistance, enhanced thermal stability, and low dielectric properties to epoxy resin while maintaining good mechanical behaviors for high-end applications, a monomer (BZPN) containing the characteristic structure of benzoxazine, phthalonitrile, and trifluoromethyl was prepared and added into the Bisphenol A-type epoxy resin (DGEBA)/Dapsone (DDS) combination. The glass transition temperature (T_g_) and carbon yield under a nitrogen atmosphere at 800 °C were found to significantly increase from 155 °C, 17.2% to 236 °C, 50.3%, respectively, for the neat EP/DDS and the BZPN-containing material. The UL-94 flammability rating achieved V-0 level when the BZPN content was 19.2 wt.% (EP-BZ-1). The thermal decomposition and flame retardancy mechanism were explored by TGA-FTIR, Raman, and XPS analysis. The fluorine-containing products were found in both the gas phase and the char residue, implying that the •CF_3_ radicals played an important role in promoting the flame-retardant behaviors through a radical trapping mechanism. The dielectric constant and dielectric loss of the materials decreased as anticipated. In addition, mechanical testing of carbon fiber-reinforced composites showed that the BZPN-containing resin presented equivalent mechanical behaviors to the neat EP/DDS resin. The synthesized BZPN was proved to be an effective and promising additive for the epoxy-based composite.

## 1. Introduction

As one of the most widely used thermosetting resins in composite materials, the improvement of epoxy resin’s (EP) comprehensive performance has become a key issue in current research and application. Some applications, such as electronic packaging, thermal interface materials, and structural composites, have stringent demands for thermal stability, flame resistance, and mechanical properties [1,2,3,4]. For example, effective flame retardancy and high thermal stability, coupled with low dielectric loss, are fundamental to prevent safety hazards leading from short circuits or overheating, and to reduce signal loss. The thermal stability and glass transition temperature (T_g_) are also critical for the structural composite of a high-speed aircraft, such as the rocket fairing, which works at a high temperature.

Diglycidyl ether of bisphenol A (DGEBA), the dominant epoxy resin accounting for over 90% of the market, faces significant application constraints due to its inherent flammability and poor thermal stability, limiting its application in the above-mentioned fields [2].

Traditionally, halogen-based compounds were used as an effective flame retardant which endowed epoxy resins with promoted flame resistance and little side effects on other properties of modified materials. However, there are strict restrictions on their application concerning the emission of hazardous corrosive gases during burning [5,6]. Phosphorus-containing compounds have been considered to be another appealing flame retardant owing to versatile reactivity, inherent safety, and superior flame suppression capabilities [7,8,9]. However, good flame-retardant behavior often brings about serious compromise on thermal stability and mechanical performance. Wang [10] modified the DGEBA with DOPO and the synthesized material was found to achieve a UL-94 V-0 classification with a phosphorus content of 5 wt.%, while resulting from the T_g_ with 16.9% reduction. Zhang’s [1] hyperbranched flame retardant (HBFR) effectively promoted the toughness and flame retardancy of DGEBA epoxy while it concurrently induced a substantial reduction in glass transition temperature. The combined enhancement, which not only promotes the flame retardancy but also the thermal stability and dielectric property, meanwhile, maintaining the mechanical performance is of great value on engineering application. The -CF_3_ radical generated from the trifluoromethylated materials can endow the materials with good flame retardancy in addition to the typical fluorine-caused properties. Du [11] synthesized a phosphorus-fluorine synergistic polymer modifier (12FOP) and used it to modify DGEBA. The modified material achieved a UL-94 V-0 rating, and its limiting oxygen index (LOI) was markedly increased. Furthermore, three-point bending tests demonstrated that the incorporation of 12FOP not only enhanced the mechanical strength of the epoxy resin (EP) but also improved its toughness.

The introduction of functional components has been proven effective in promoting the comprehensive performance of epoxy resin. Phthalonitrile, known for its excellent thermal stability after curing, has been extensively studied and widely applied in advanced fields. The curing reaction generates triazine rings and phthalocyanine rings with high stiffness, imparting the material with exceptional thermal stability, mechanical properties and flame retardancy. Xu et al. [12] reported an epoxy/phthalonitrile pre-polymers (APPEN). The APPEN/GF composites demonstrated exceptional mechanical performance, with a flexural strength of 712 MPa and modulus of 38 GPa, while maintaining high thermal stability (T_g_ > 185 °C). Hu et al. [13] developed binary blends of benzimidazole-functionalized phthalonitrile (PNBI) with DGEBA. Dynamic mechanical analysis revealed glass transition temperatures (T_g_) exceeding 207 °C for the cured blends. Thermogravimetric analysis of the nitrogen atmosphere demonstrated exceptional thermal stability, exhibiting T_5_% > 360 °C and a char yield exceeding 14% at 800 °C. Nevertheless, novel challenges have emerged, including poor compatibility between the additive agent and the base material, and a weak interface between the base material and the functional components, which are likely to negatively influence the matrix. To solve these problems, building a cohesive interface between the two components has proved to be effective [14,15,16]. Benzoxazine resin, with the characteristic oxazine ring, has been attracting interest due to its design flexibility and many outstanding behaviors such as high dimensional stability, good thermal stability, and dimensional stability during curing [17,18,19,20]. Moreover, the generated phenolic hydroxyls can polymerize with epoxy groups during the heating process, forming covalent bonding with the epoxy matrix.

In the current work, a new monomer combining the typical groups of phthalonitrile, benzoxazine, and trifluoromethyl was prepared and used as the functional additive for the typical epoxy resin, i.e., the DGEBA. The incorporation of this novel flame retardant simultaneously enhanced flame resistance, thermal stability, and dielectric performance. The purpose of this paper is to present a practical and feasible way to concurrently promote the above-mentioned behaviors of epoxy resin, maintaining a good mechanical performance.

## 2. Experiment

### 2.1. Experimental Materials

The bisphenol-A epoxy resin (DGEBA) (E_w_: 0.57–0.61) was from Blue Star Chemical New Materials Co., Ltd. (Beijing, China); Hexafluorobisphenol A (BPAF), 4,4-Diaminodiphenylsulfone (DDS), 3,4-Dicyano-1-nitrobenzene (NPh), N, N-dimethyl formamide (DMF), anhydrous carbonic acid, dipotassium salt (K_2_CO_3_), sodium hydroxide, p-aminophenol and polyformaldehyde were from Aladdin Biochemicals Technology Co., Ltd. (Shanghai, China); All reagents were of analytical grade and used as received without further purification. The plain weave carbon fiber orthogonal fabric (T-300, 3K) with areal density of 300 g/m^2^ was supplied by Weihai Guangwei Composite Materials Co., Ltd. (Weihai, China).

### 2.2. Synthesis of 4-APN

NPh (17.3 g, 100 mmol), p-aminophenol (10.9 g, 100 mmol), anhydrous K_2_CO_3_ (17 g, 120 mmol) were sequentially mixed into the flask containing 50 mL of DMF with continuous stirring. The reaction mixture was held at 80 °C under a nitrogen environment for a duration of 8 h. Following the completion of the reaction, the anhydrous K_2_CO_3_ was removed by filtration. The filtered solution was slowly added to the 1N NaOH solution, leading to the formation of a yellow-brown precipitate. The resulting solid was then washed with deionized water and dried under a vacuum at 80 °C for 24 h, yielding 4-APN with 88.6%.

### 2.3. Synthesis of BZPN

**Figure 1:**
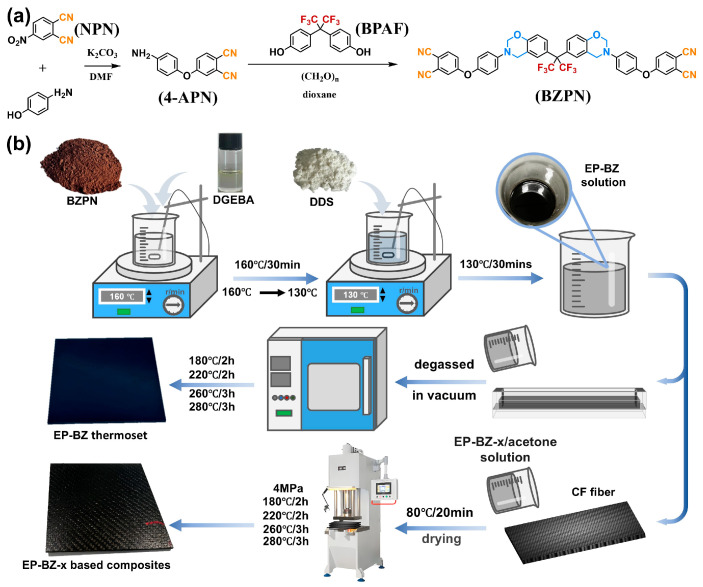
(**a**) Synthesis pathway of the BZPN, (**b**) Preparation flow chart of EP-BZ thermosets and EP-BZ-x based composites.

The benzoxazine monomer in this article was synthesized through the Mannich reaction among primary amine, phenol and aldehyde. In this paper, the synthesized 4-APN [21] and BPAF were used as the primary amine and phenol, respectively, to synthesize the benzoxazine/phthalonitrile hybrid monomer BZPN, as shown in Figure 1a. Specifically, 47 g of 4-APN, 12 g of paraformaldehyde, and 80 mL of 1,4-dioxane were sequentially mixed under continuous stirring. The temperature was kept low via ice bath for 2 h. Subsequently, BPAF (33.6 g, 100 mmol) was introduced into the reaction mixture, and the temperature was maintained at (90 ± 5) °C under reflux conditions for 8 h. The BZPN solution was poured into cold water, obtaining the precipitated BZPN. After being vacuum-dried the final BZPN was obtained yielding 78.2%.

### 2.4. Preparation of the Epoxy-Based Thermosets

In this study, DGEBA was cross-linked using DDS. The epoxy system (i.e., the control group) was prepared by mixing DGEBA with DDS at (130 ± 5) °C with continuous stirring for (18 ± 2) minutes until complete dissolution. The homogeneous mixture was cured using a two-stage procedure: 160 °C/2 h + 180 °C/4 h. This control formulation was designated as EP-BZ-0 for subsequent characterization. For the specimens containing DGEBA, DDS and BZPN, the curing process was as follows: DGEBA was first mixed with BZPN at (160 ± 5) °C under continuous stirring until BZPN was fully dissolved. The mixture was subsequently cooled to the temperature of (130 ± 5) °C, before DDS was introduced. The curing process was carried out sequentially at 180 °C for 2 h, followed by 220 °C for 2 h, then 260 °C for 3 h, and finally 280 °C for 3 h. The specimens were designated as EP-BZ-x, in which ‘EP’ denotes the DGEBA/DDS moiety, ‘BZ’ represents BZPN, and ‘x’ represents the amount of BZPN. The specific compositions of the specimens are presented in Table 1.

### 2.5. Preparation of the EP-BZ-x Based Composites

Carbon fiber (CF) fabric was impregnated with a 30 wt.% EP-BZ-x/acetone solution, followed by vacuum solvent removal in 80 °C for 1 h. Ten layers of the prepregs were stacked in a stainless steel mold and cured in hot press (FCC-D350, Yiqing Machinery Co., Ltd., Wuxi, China) with pressure of 4 MPa and the curing process was conducted in a stepwise manner: 180 °C for 2 h, followed by 220 °C for 2 h, then 260 °C for 3 h, and finally at 280 °C for 3 h. The mass ratio of matrix to CF was about 4:6. The preparation process was shown in schematic Figure 1b.

### 2.6. Characterization

NMR spectra were obtained using a Bruker Avance NEO 400 MHz spectrometer (Bruker BioSpin GmbH, Rheinstetten, Germany).

FTIR spectra were obtained using a Thermo Fisher Nicolet iS10 spectrometer (Thermo Fisher Scientific, Waltham, MA, USA) with the KBr technique, covering a wavenumber range of 4000 to 400 cm^−1^. The measurements were performed with 8 scans and resolution of 4 cm^−1^.

Thermogravimetric analysis (TGA) was performed using a Mettler TGA2 instrument (Mettler-Toledo International Inc., Greifensee, Switzerland) under nitrogen atmosphere with flow rate of 50 mL/min. The temperature was increased from 50 to 800 °C, with a heating rate of 10 K/min. For each sample, three individual specimens were tested as part of the standard procedure, and the average result was reported.

Differential scanning calorimetry (DSC) was recorded on a Mettler DSC 3+ analyzer (Mettler-Toledo International Inc., Greifensee, Switzerland) with N_2_ purge (50 mL/min), scanning 50–400 °C with heating rates of 5–20 K/min. Peak deconvolution was performed using Origin 2021 with Gaussian-Lorentz Cross mode.

Dynamic mechanical analysis (DMA) was performed using the DMA 242E (Netzsch) instrument (NETZSCH-Gerätebau GmbH, Selb, Germany) operating in dual-cantilever mode. The measurements were carried out at a frequency of 1 Hz and a heating rate of 5 K/min.

The Limiting Oxygen Index (LOI) was determined using an HC-2C instrument (Nanjing Shangyuan Analytical Instrument Co., Ltd, Nanjing, China) in accordance with ASTM D2863 [22]. The test specimens used had dimensions of 130 mm × 6.5 mm × 3.0 mm.

Cone calorimetry (CONE) tests were performed on a FTT iCone Classic (Fire Testing Technology (FTT), West Sussex, UK) with irradiance of 35 kW/m^2^ and specimens of 100 mm × 100 mm × 3 mm.

UL-94 tests were conducted on M607B equipment (Qingdao ruixinjie Instrument Co., Ltd, Qingdao, China) according to ASTM D3801 [23] with specimens of 125 mm × 13 mm × 3.5 mm.

Scanning electron microscopy (SEM) analysis was conducted using a JSM-7500F microscope (JEOL Ltd., Tokyo, Japan), operated at an accelerating voltage of 5 kV. The instrument was fitted with an X-Max N80 energy dispersive X-ray spectrometer (EDS) (Oxford Instruments, Oxfordshire, UK) for elemental analysis.

Raman spectroscopy was carried out using a Thermo Fisher (DXR, Waltham, MA, USA) laser confocal micro-Raman spectrometer, employing a laser with a wavelength of 532 nm.

X-ray photoelectron spectroscopy (XPS) measurements were performed on an ESCALAB 250 instrument (Thermo Fisher Scientific, Waltham, MA, USA) equipped with an Al Kα X-ray source.

Thermogravimetric analysis coupled with infrared spectroscopy (TG-IR) was conducted using a TA Q50 thermogravimetric analyzer (TA Instruments, New Castle, DE, USA), connected to a Thermo Fisher infrared spectrometer under a nitrogen atmosphere. A transfer line maintained at 200 °C was used to interface the TGA and FTIR systems.

The dielectric properties of the carbon fiber reinforced polymer (CFRP) were assessed, employing an E4991B impedance analyzer (Keysight Technologies, Santa Rosa, CA, USA).

Interlaminar shear strength (ILSS) was assessed using the short beam method as specified in ASTM D2344 [24]. Flexural strength testing was carried out in accordance with ASTM D790 [25] at a crosshead speed of 1 mm/min. All mechanical test results were averaged over five individual samples.

## 3. Results and Discussion

### 3.1. Structural Characterization of BZPN

**Figure 2:**
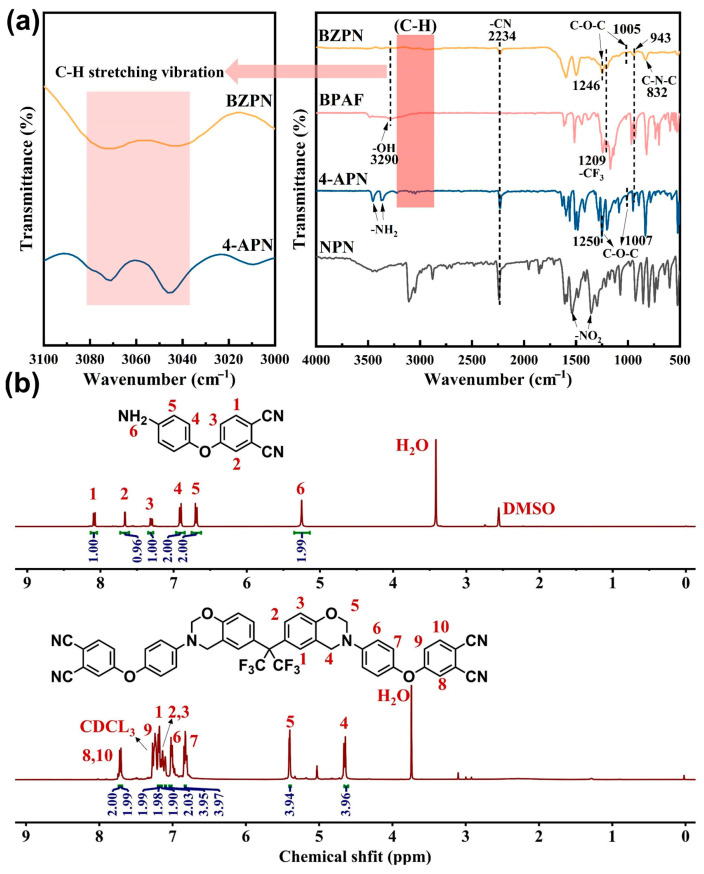
(**a**) FT-IR analysis and (**b**) 1H NMR spectra characterization of the BZPN.

The molecular structure of the synthesized BZPN was confirmed using FT-IR and NMR (^1^H) techniques, with the results presented in Figure 2a,b. In the FTIR spectrum, the characteristic absorption band corresponding to the phenolic hydroxyl group in BPAF appears at approximately 3290 cm^−1^. Additionally, the signals attributed to the -NH_2_ group in 4-APN, observed at 3372 cm^−1^ and 3454 cm^−1^, are no longer present in the BZPN spectrum. Furthermore, absorption peaks associated with the cyano and trifluoromethyl groups are detected at 2234 cm^−1^ and 1209 cm^−1^, respectively, confirming their incorporation into the BZPN structure [26]. The signals of benzoxazine at 943 cm^−1^ (trisubstituted benzene ring attached to oxazine ring), C-O-C at 1005 cm^−1^ and 1246 cm^−1^ (symmetric and asymmetric stretch), C-N-C at 832 cm^−1^ [27,28], and the signals at 3045 cm^−1^ 3071 cm^−1^ (symmetric and antisymmetric stretching of C-H in the benzene ring) [29,30] are observed in the spectrum of BZPN.

^1^H NMR of 4-APN (400 MHz, DMSO-d6): δ = 8.15 (d, J = 8.7 Hz, 2H), 7.94 (d, J = 2.6 Hz, 2H), 7.54 (dd, J = 8.7, 2.6 Hz, 2H), 7.49 (d, J = 8.5 Hz, 4H), 7.35–7.28 (m, 4H) ppm. Preparation process was shown in Figure 1a.

^1^H NMR of BZPN (400 MHz, CDCl_3_) δ 7.72 (d, J = 8.2 Hz, 2H) 7.69 (d, J = 8.4 Hz, 2H), 7.26–7.23 (m, 2H), 7.22–7.20 (m, 2H), 7.18–7.16 (m, 2H), 7.09–7.08 (m, 2H), 7.01–6.98 (m, 4H), 6.83–6.80 (m, 4H), 5.39 (d, J = 3.2 Hz, 4H), 4.63 (d, J = 7.6 Hz, 4H). Preparation process was shown in Figure 1a.

### 3.2. Curing Study

**Figure 3:**
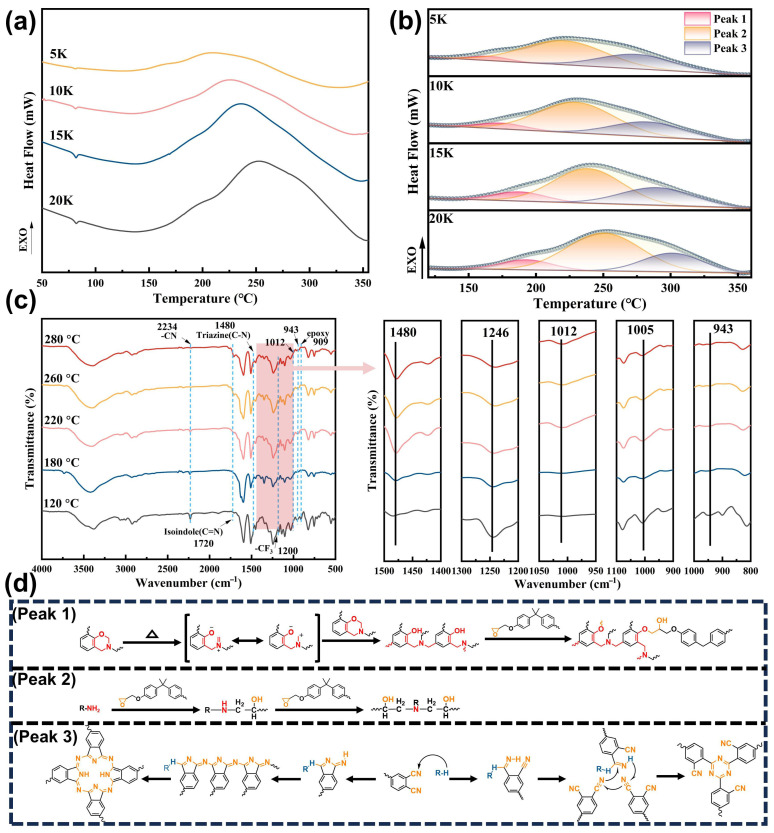
Curing study of the EP-BZ-3 blend: (**a**) Non-isothermal DSC curves of the curing process, (**b**) deconvolution of the DSC curves, (**c**) in situ FT-IR study of the curing process, (**d**) the schematic diagram of the curing mechanism.

Curing process of the EP/DDS/BZPN blend (i.e., the EP-BZ-3) was monitored with DSC and the original DSC curves (Figure 3a) were deconvoluted as shown in Figure 3b. The original exothermic peak can be well deconvoluted into three individual peaks. The peak at a relatively low temperature (peak 1) can be attributed to the ring-opening of oxazine ring. The biggest exothermic peak (peak 2) can be assigned to the reaction between the epoxy group in DGEBA and the primary amino in DDS, according to the previous study [31,32,33]. The exothermic peaks at high temperature (peak 3) can be attributed to the polymerization of phthalonitrile, during which the typical phthalocyanine, triazine and isoindole structures were formed. The polymerization progression was investigated via FTIR, as depicted in Figure 3c. The characteristic signal of epoxide functional group at 909 cm^−1^ disappears after treating at 180 °C, which indicates that BZPN has no inhibition on the crosslink of EP/DDS. The characteristic signals of benzoxazine at 1246 cm^−1^ and 1005 cm^−1^ (stretch, C-O-C) decreased obviously after heating at 180 °C, indicating the ring-opening of the oxazine ring. The peak at 943 cm^−1^ gradually decreased with the curing process, which confirmed the crosslink of the opened oxazine ring. The characteristic peak of the cyano group (2234 cm^−1^) in BZPN gradually decreases after the temperature reaches 220 °C, and completely disappears after being treated in 280 °C. Simultaneously, the characteristic signals at 1720 cm^−1^, 1480 cm^−1^ and 1012 cm^−1^ [34,35] which correspond to isoindole, triazine and phthalocyanine rings, respectively, were observed after the specimens were treated at 220 °C. The results of FTIR characterization were consistent with the DSC data. Thus, the curing mechanism can be confirmed as follows: At the initial heating stage, the oxazine ring opened and formed the phenolic hydroxyl. The ring-opening reaction of oxazine was followed closely by the crosslinking reaction of EP/DDS. Meanwhile, the generated phenolic groups can react with epoxy groups [36,37] to form a covalent bond between the EP/DDS network and the polymerized BZPN. At relatively high temperature, the polymerization of the cyano group in BZPN took place and the stable cyclic structures (i.e., phthalocyanine, triazine and isoindole rings) were formed. The specific reactions are depicted in Figure 3d.

### 3.3. Thermal Stability

**Figure 4:**
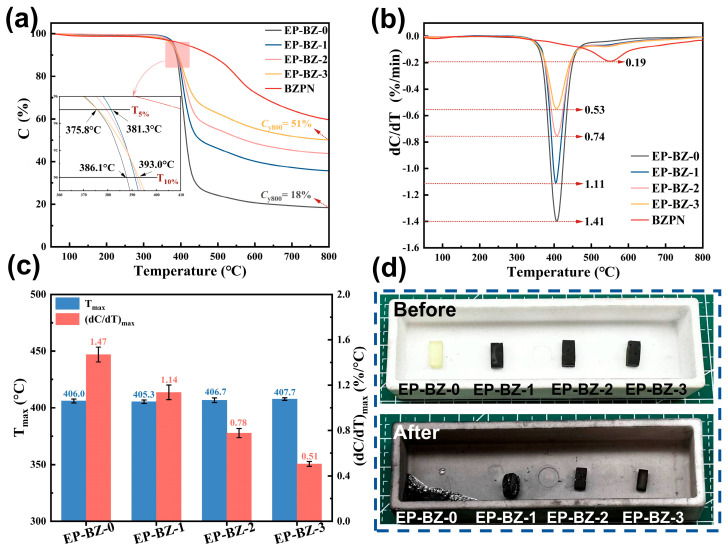
(**a**) TGA and (**b**) DTG curves of the TGA test and (**c**) values of T_max_ and (dC/dT)_max_ (**d**) photograph of cured specimens before and after pyrolysis under air condition at 500 °C.

The pyrolysis resistance is the key reflection of the thermal stability of polymers. As demonstrated in Figure 4a,b, the incorporation of BZPN significantly increased the final char yield (C) and reduced the maximum rate of weight loss ((dC/dT)_max_). Additionally, BZPN notably enhanced char formation and inhibited the degradation rate of the materials. The char yield at 800 °C/N_2_ increased from 17.2% to 50.3% and the maximum weight losing rate decreased from 1.47 to 0.51, respectively, for neat EP/DDS and EP-BZ-3.

The char yield and mass loss rate of a blended material at a given temperature can be theoretically calculated based on Equations (1) and (2), under the assumption that each constituent undergoes decomposition independently and without chemical interaction.

(1) $$C=\left(C_{1}\times w_{1}+C_{2}\times w_{2}\right)\times 100\%$$

(2) $${\left.\frac{dC}{dT}\right|}_{T_{1}}=w_{1}{\left.\frac{{dC}_{1}}{dT}\right|}_{T_{1}}+w_{2}{\left.\frac{{dC}_{2}}{dT}\right|}_{T_{1}}$$

Herin, C represents the char yield of the composite material, C_1_ and C_2_ denote the char yields of the individual components, w_1_ and w_2_ correspond to their respective mass proportions and T denotes the temperature.

The char yield at 800 °C and the peak dC/dT values (i.e. (dC/dT)_max_) were determined using Equations (1) and (2), as presented in Table 2. All experimentally obtained char yields exceeded the theoretical values, whereas the observed (dC/dT)_max_ values were comparatively lower. The discrepancies between the experimental and calculated results suggest that the incorporated BZPN may exert an inhibitory influence on the thermal decomposition of the DGEBA/DDS structure. Additionally, the covalent linkage formed between the epoxy functional group and the oxazine ring (illustrated in Figure 3d) could be associated with this observed effect.

It should be noted that the initial and maximum pyrolysis temperature of the materials were not affected much by the BZPN. The T_5_%, T_10_% and T_max_ values of the BZPN-containing materials are very close to that of neat epoxy resin (i.e., EP-BZ-0). The result implies that the major structure of the material is not changed by the introduction of BZPN (still be the epoxy/amine crosslinking structure), while the maximum weight losing rate was effectively suppressed, and decreased from 1.41%/min to 0.53%/min as shown in Figure 4b,c. To give the most intuitive reflection on the thermal stability of the materials, the specimens with a similar appearance were heated in tubular furnace in air atmosphere to 500 °C. The original and final conditions are shown in Figure 4d. The pure EP-BZ-0 showed a totally collapsed profile, while the chars for EP-BZ-2 and EP-BZ-3 maintained the identical profiles to the original specimens. The results demonstrated that BZPN are highly effective at restraining the thermal decomposition in the epoxies.

The T_g_ and the modulus at elevated temperatures are also key parameters for evaluating structural materials intended for high-temperature applications. In this study, the T_g_ values were determined based on the peak temperatures of tangent δ obtained from dynamic mechanical analysis (DMA) as shown in Table 3.

**Figure 5:**
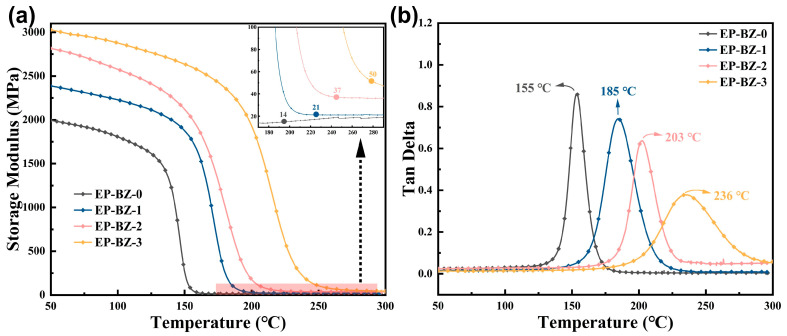
The storage modulus curves (**a**) and tangent δ plots (**b**) of thermosets from DMA tests.

As shown in Figure 5b the T_g_ of the cured resin is obviously and regularly promoted by the addition of BZPN. The T_g_ value significantly increased from 155 °C (EP-BZ-0) to 235 °C (EP-BZ-3). Moreover, the addition of BZPN effectively promoted the E′ value at rubber state (i.e., at T_g_ + 40 °C) as shown in Figure 5a. The E′ of EP-BZ-3 at rubber state (50 MPa) increased by 257% compared to that of EP-BZ-0 (14 MPa). The E′ at glassy state also obviously increased with the increase in BZPN. It is noteworthy that, in our previous work, we found that the E′ of glassy state of the cured resin containing the similar phthalonitrile monomer (AFPN) without oxazine group was not obviously promoted. The phenolic hydroxyl, which was generated during the ring opening of oxazine groups, could undergo addition polymerization with the epoxy groups, leading to the formation of covalent bond. The increased E’ of glassy state can be attributed to the formed covalent bonding between the rigid structures formed by the phthalonitrile and the crosslinked EP/DDS network.

### 3.4. Flame Resistance

**Figure 6:**
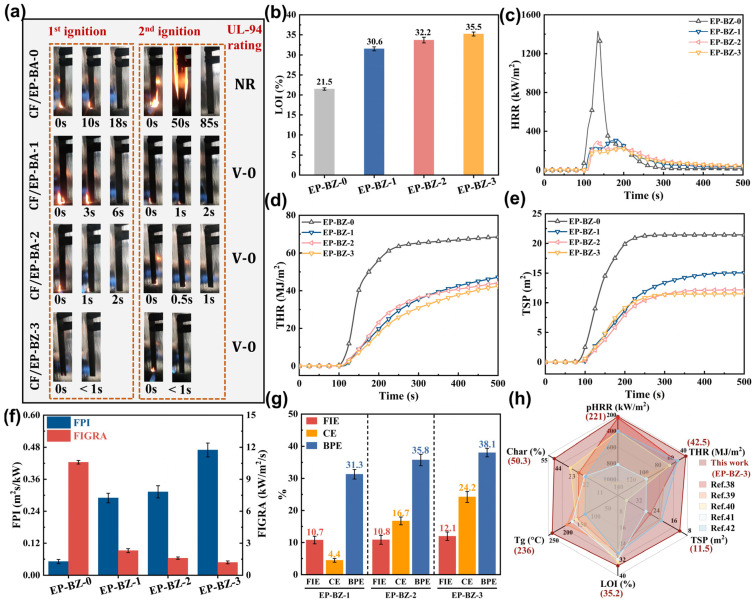
(**a**) Photographs of the carbon fiber composite specimens during the UL-94 tests (**b**) LOI values (**c**) HRR, (**d**) THR, (**e**) TSP curves as function of time; The calculated values of (**f**) FPI, FIGRA, (**g**) FIE, CE, BPE and (**h**) the comparison of major behaviors with relevant references [38,39,40,41,42].

The flame resistance of polymeric materials, particularly those utilized in high-temperature environments, is a crucial factor in ensuring safety during application. The photos of the CF composites during the UL-94 tests are shown in Figure 6a. As it is well-known, the neat EP/DDS is combustible during the test. There was significant promotion of the flame retardancy of the material under the small load of BPZN. The CF/EP-BZ-1 reached V-0 level which self-extinguished within 6 s during the 1st ignition. The CF/EP-BZ-3 became incombustible with an extinguishing time of less than 1 s. The LOI of the thermosets increased from 21.5 to 35.5, respectively, for neat EP-BZ-0 to EP-BZ-3.

The flame retardancy behaviors were further studied by the CONE and the results are shown in Table 4. Figure 6c–e presents the fundamental data for the heat release rate (HRR), total heat release (THR), and total smoke production (TSP). The combustion process of the materials demonstrated a significant reduction in both heat release and smoke generation. The peak HRR (pk-HRR) value significantly decreased from 1431 kw/m^2^ to 221 kw/m^2^, for EP-BZ-0 to EP-BZ-3, respectively.

Some further quantitative indicators can be obtained from the original Cone data. The fire performance index (FPI), defined as TTI (time to ignition) divided by the peak heat release rate (pk-HRR), and the fire growth rate (FIGRA), calculated as pk-HRR divided by the time to reach pk-HRR (TPHRR), are presented in Figure 6f. An increased FPI suggests a reduced fire hazard, while a decreased FIGRA reflects a slower development of the fire. It can be concluded that the increase/decrease on FPI/FIGRA of EP-BZ-2 and EP-BZ-3 to EP-BZ-1 was not as obvious as that of EP-BZ-1 to EP-BZ-0, which was consistent with the results in LOI and UL-94 tests. In other words, only a small amount of BZPN is needed to provide a significant promotion of the flame-retardant performance of the epoxies.

Herein, to further study the influence of BZPN content on the flame resistance, another three indicators were used: (1) flame inhibition effect (FIE); (2) the charring effect (CE); and (3) barrier and protective effect by char layer (BPE). The FIE can be quantitatively calculated using the effective heat combustion (EHC) value: FIE = 1 − EHCFREP/EHCEP. The CE can also be calculated using the char yield values: CE = 1 − (1 − CharFREP)/(1 − CharEP). The decreased degree of pk-HRR of BPZN-containing samples were much higher than that of THR as shown in Figure 6c,d. The mismatched decrease can be attributed to the barrier and protective effect by the char layer and calculated as: BPE = 1 − (pk − HRRFREP/pk − HRREP)/(THRFREP/THREP). The calculated FIE, CE and BPE values are shown in Figure 6g. It can be found that the growth trend of CE with increased BZPN loading was more obvious than those of FIE and BPE. In other words, a small amount of BZPN can provide an obvious flame-retardant effect and the charring effect will be the dominant factor for the further increase in flame-retardant behavior by increasing the BZPN loading.

The measured values of Tg, LOI, THR, TSP, peak HRR, and char yield for EP-BZ-3 were compared with data from the relevant literature, as illustrated in Figure 6h [38,39,40,41,42]. The EP-BZ thermosets presented in this study offer a distinct advantage as additives for epoxy materials, enhancing both thermal stability and fire-retardant performance.

### 3.5. Flame Retardancy Mechanism

#### 3.5.1. Condensed Phase

**Figure 7:**
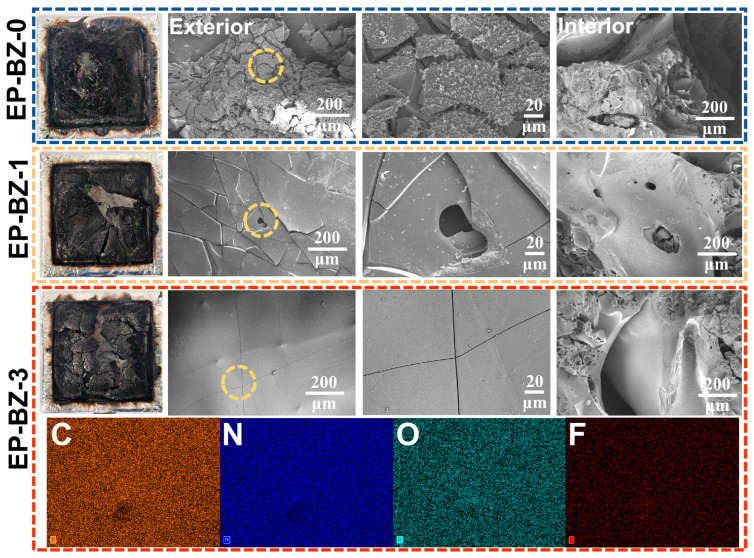
Digital images and scanning electron micrographs of carbon yield for the thermosets and EDS element mapping for carbon yield of EP-BZ-3.

The average char yields of the cured EP-BZ-0, EP-BZ-1 and EP-BZ-3 after CONE tests were 13.9%, 37.5% and 42.6%, respectively. Both the internal and external surfaces of the char of EP-BZ-1 and EP-BZ-3 were smoother and denser compared with that of EP-BZ-0, as shown in Figure 7, directly confirming the promoted charring effect (CE) and barrier/protective effect (BPE) discussed above. Furthermore, the EDS mapping of EP-BZ-3 reveals a homogeneous dispersion of fluorine, indicating that BZPN is evenly distributed within the epoxy matrix.

**Figure 8:**
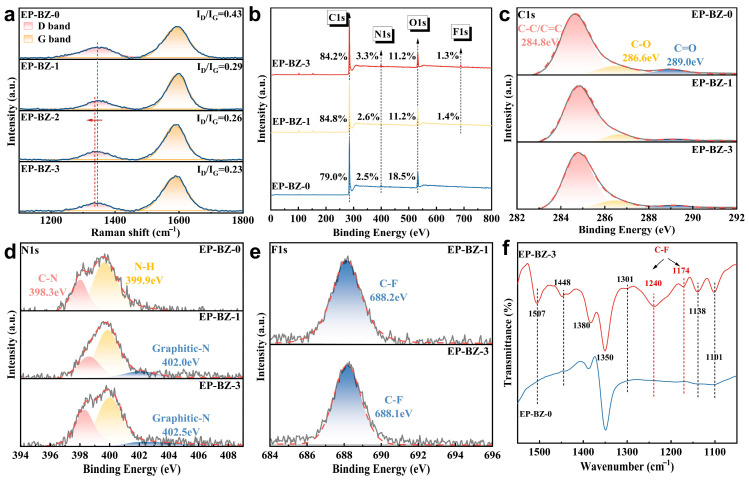
(**a**) Raman spectra, (**b**) XPS full spectra, and high resolution XPS spectra of (**c**) C1s (**d**) N1s (**e**) F1s and (**f**) FTIR spectra of the char residues.

The formed compact carbon residue plays as the protective barrier, shielding the underlying materials and inhibiting the heat/mass transmission. As shown in Figure 8a, the carbon residue was further analyzed through Raman spectroscopy. The Raman absorption peaks at 1350 cm^−1^ (D band) and 1580 cm^−1^ (G band) are two important indicators for structure characterization of carbon-based materials. It is noted that there is a red shift for the D band, which may be caused by the formation of nitrogen-doped graphite structure (since the doped N atom will cause the lattice distortion and lead to the increase in structural tension). The I_D_/I_G_ ratio, derived from the integrated areas of the D and G bands, is commonly utilized as an indicator to evaluate the structural graphitization. The high I_D_/I_G_ indicates a high degree of defect or disorder of the structure (such as graphene oxidation, mechanical damage). On the contrary, a low I_D_/I_G_ means a high degree of graphitization and integrity [43,44]. The I_D_/I_G_ values regularly decreased from 0.43 to 0.23, with the increase in BZPN loading. The result indicates that the introduction of BZPN is favorable to the graphitization of the char during combustion. The graphitization tends to form the char with a more compact and denser structure, which is favorable to the barrier and protective effect.

Figure 8b shows the overall XPS spectra of the char residues. Fluorine element was found at the surface of the char of EP-BZ-1 and EP-BZ-3, which implied that the •CF_3_ radicals took part in the pyrolysis and rearrangement process in the condensed phase. The deconvoluted high resolution XPS spectra are shown in Figure 8c–e. Decrease for the peak at 289 eV, which belongs to C=O bonding, can be observed [45,46]. This confirms that the oxidation reaction was suppressed. It shows that the residual carbon has a certain blocking effect on external oxygen. In the N1s spectra, the fitted peaks at 402 eV, which are attributed to graphitic-N structure, are observed in the spectra of EP-BZ-1 and EP-BZ-3 [39]. The N-doped structure was proved to have lower combustion enthalpy compared to the undoped ones [47], leading to a significant increase in the thermal stability against combustion. Therefore, this compact graphite-like structure can significantly suppress the emission of gases and heat, ultimately offering enhanced protection to the matrix [48]. It is worth noting that the F1s binding energy was consistently observed at 688.2 eV, as depicted in Figure 8e, which implied that the F existed in the form of CF_3_. The FTIR spectra of the chars within the fingerprint area were shown in Figure 8f. The spectrum of EP-BZ-3 is more complex with peaks at 1507 cm^−1^, 1448 cm^−1^, 1301 cm^−1^, 1240 cm^−1^, 1174 cm^−1^, 1138 cm^−1^, 1101 cm^−1^ which are not observed in that of EP-BZ-0. Besides the peaks at 1240 cm^−1^ and 1174 cm^−1^ (asymmetric and symmetric stretch of C-F), the others can be attributed to the structures of nitrogen-doped heterocycle or fused ring, such as pyridine, quinoline, and pyrrole [49,50,51]. It implies that the nitrogen-doped structures of the char, similar to the above-mentioned nitrogen-doped graphite, exert a critical influence on the improvement of the thermal and flame-retardant behaviors.

#### 3.5.2. Gas Phase

**Figure 9:**
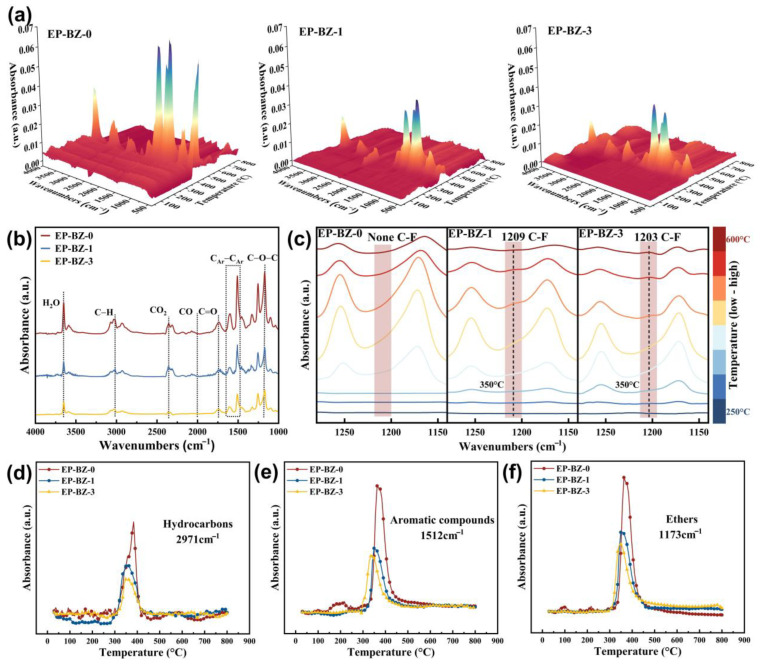
(**a**) 3D TG-IR spectra of EP-BZ-0, EP-BZ-1, EP-BZ-3 and (**b**) FTIR spectra of gaseous products at the peak mass-losing rate temperature and (**c**) the characteristic absorption of C-F bond at different temperatures. The temperature-dependent absorbance of typical gaseous products for (**d**) hydrocarbons, (**e**) aromatic compounds, and (**f**) ethers.

TG-IR spectroscopy was used to investigate the pyrolysis gas of the thermosets. As shown in Figure 9a–c, a reduction in the absorbance of gaseous products is evident as the BZPN content increases, suggesting that BZPN significantly inhibits the generation of volatile gaseous during decomposition. The FT-IR spectra obtained at the point of the maximum decomposition rate are displayed in Figure 9b. The spectra revealed similar gaseous products for neat EP and the BZPN-containing ones, except for the signal intensity, which suggests that the crosslinked BZPN moiety suppressed the evolution of gaseous pyrolyzates under thermal degradation. It is noteworthy that C-F signal at 1209 cm^−1^ became detectable at an onset temperature of 350 °C, which implied the generation and recombination of the •CF_3_ radicals during the combustion.

The temperature-dependent absorbance of typical degradation products, including hydrocarbons (3016 cm^−1^), aromatic compounds (1508 cm^−1^), and ethers (1168 cm^−1^), are shown in Figure 9d–f. The gas generations of the BZPN-containing specimens were obviously lower than that of EP-BZ-0, confirming the effective influence on gas phase flame retardancy. It is important to find that the temperatures corresponding to the peaks in Figure 9d–f are very close to the temperature at which the C-F signals occur. Thus, it can be concluded that the formation of fluorine-containing radicals is closely linked to the enhancement of flame retardancy in the gas phase.

**Figure 10:**
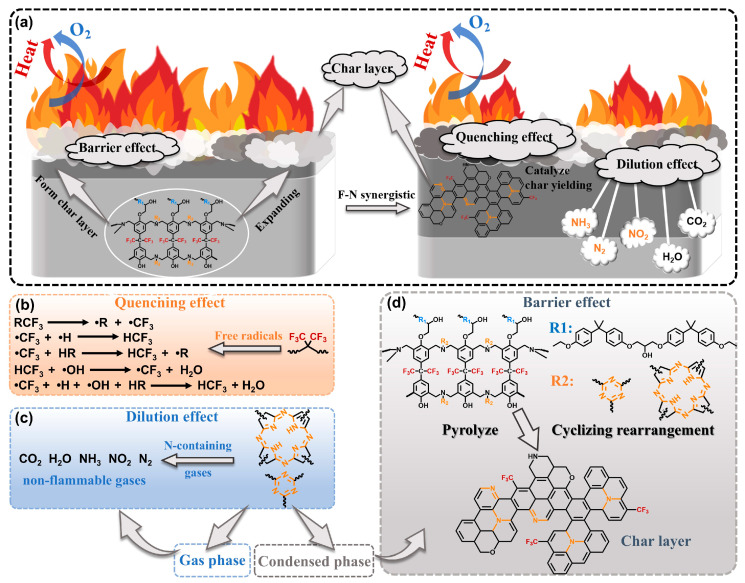
Flame-retardant mechanism diagram of BZPN-containing materials: (**a**) the schematic diagram of flame-retardant process, (**b**) quenching effect, (**c**) dilution effect, and (**d**) barrier effect. Reproduced from ref. [52] with permission from the Elsevier, copyright 2024.

The improved combustion resistance of the BZPN-containing thermoset resulted from both the condensed-phase and gas-phase effects, as illustrated in schematic Figure 10: (1) The formed phthalocyanine, triazine, and isoindole structures from the BZPN undergo cleavage and rearrangement reactions, forming the graphite-like nitrogen-rich carbon residue which acts as the barrier restricting heat and mass transfer, protecting the underlying polymer matrix by isolating it from external oxygen and heat sources. (2) The •CF_3_ free radicals generated during thermal degradation efficiently capture reactive radicals produced in the decomposition process, thereby interrupting the combustion chain reaction. Moreover, the enhanced thermal stability of the EP-BZ-x thermoset effectively reduces the release of flammable gases, such as carbonyl, aromatic, and ether compounds. Overall, the improved fire resistance of the thermoset arises from the combined action of the phthalonitrile and trifluoromethyl groups.

### 3.6. Mechanical and Dielectric Properties of Carbon the CF Composites

Carbon fiber (CF) reinforced composites were prepared and the mechanical and dielectric behaviors were tested. The flexural strength and modulus showed an upward trend with the increase in BZPN content, due to the increased rigid and crosslink density of the structure. The ILSS is influenced by both the strength of the matrix and the interfacial strength between the fiber and the matrix. Although the strength of the matrix increased, the interfacial strength is likely to be negatively influenced by the BZPN, since the commercial sizing agent was not well fitted to the BZPN and the relevant high curing temperature. As a result, the ILSS values showed the up–down trend with the increasing BZPN content.

**Figure 11:**
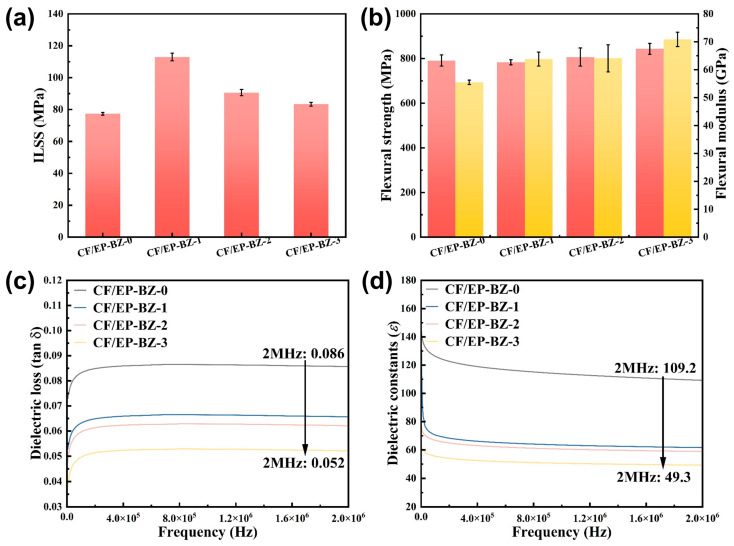
(**a**) ILSS values, (**b**) flexural strength and modulus, (**c**) dielectric constant and (**d**) dielectric loss values of the composites.

The dielectric constant (ε) and loss tangent (tan δ) critically govern electromagnetic wave transmission. Low-ε matrix composites reduce signal attenuation and distortion in high-frequency applications. As illustrated in Figure 11c,d, both the dielectric constant (ε) and the loss tangent (tan δ) show a significant reduction as the BZPN content increases. This decline in dielectric properties can be explained by the larger free volume and reduced polarization capability associated with the C–F bond [26,34].

## 4. Conclusions

A novel monomer incorporating the structural features of benzoxazine, phthalonitrile, and trifluoromethyl groups (BZPN), serving as a reinforcing additive for EP matrix, has been developed and reported. The influence and mechanism of BZPN on the promotion of flame resistance and thermal stability was investigated. It was found that low addition of BZPN brought in an obvious promotion of the flame-retardant behaviors of the cured resin (with 19.2 wt.% BZPN loading, UL-94 V0, LOI: 30.6). The flame-retardant effect is derived from free radical capture by •CF_3_ radicals and the protective effect by the enhanced char formation. The other key properties such as the T_g_, char yield, and the maximum decomposition rate were also effectively enhanced. Moreover, it was confirmed that the mechanical behaviors of the CF-reinforced composites containing the BZPN showed no obvious deterioration. The dielectric constant and dielectric loss were found to decrease as expected. The BZPN monomer reported in this research was proven to be a promising strengthening additive for the epoxy-based composites calling for high performance, especially in flame retardancy and thermal stability.

## Figures and Tables

**Table 1:** Formulation of the thermosets.

Sample	DGEBA (g)	DDS (g)	BZPN (g)	BZPN Content (wt.%)	F Content (wt.%)
EP-BZ-0	10	3.0	0		
EP-BZ-1	10	2.6	3.0	19.2%	2.5%
EP-BZ-2	10	2.3	5.0	29.0%	3.8%
EP-BZ-3	10	2.0	7.0	36.8%	4.9%

**Table 2:** TGA results of the cured resins at N_2_ atmosphere.

Sample	^a^ T_5%_ (°C)	T_10%_ (°C)	T_max_ (°C)	^b^ (dC/dT)_max_ (%/K)		^c^ C_800_ (%)	
				Measured	Calculated	Measured	Calculated
BZPN	411.0 ± 1.6	481.6 ± 2.0	551.4 ± 2.3	−0.19 ± 0.05	/	62.3 ± 2.2	/
EP-BZ-0	375.8 ± 2.2	386.1 ± 1.6	406.0 ± 1.7	−1.47 ± 0.07	/	17.2 ± 1.5	/
EP-BZ-1	379.5 ± 1.8	390.5 ± 1.0	405.3 ± 1.5	−1.14 ± 0.06	1.15	35.1 ± 1.5	30.8
EP-BZ-2	381.3 ± 1.4	392.0 ± 1.7	406.7 ± 2.1	−0.78 ± 0.04	1.01	42.9± 1.8	39.2
EP-BZ-3	379.5 ± 2.6	392.6 ± 2.4	407.7 ± 1.2	−0.51 ± 0.02	0.9	50.3 ± 1.2	47.5

Note: ^a^ Temperature corresponding to 5% mass loss, ^b^ maximum mass-losing rate, ^c^ char yield at 800 °C.

**Table 3:** DMA results for the thermosets systems.

Sample	E′ at 50 °C (MPa)	E′ at (T_g_ + 40) °C (MPa)	T_g_ (°C)	*ν*_e_ ^a^ (mol/cm^3^)
EP-BZ-0	2006	14	155	1199
EP-BZ-1	2386	21	184	1690
EP-BZ-2	2815	37	203	2874
EP-BZ-3	3028	50	236	3651

Note: ^a^ The *ν*_e_ values were calculated according to the classical formula: *ν*_e_ = E′/3RT. E′ denotes the storage modulus of the material measured at temperature T, R represents the gas constant (8.314 J/K/mol), T is defined as T_g_ + 40 K.

**Table 4:** Cone data of the thermosets.

Sample	TTI (s)	pk-HRR ^a^ (kW/m^2^)	THR (MJ/m^2^)	pk-SPR ^b^ (m^2^/s)	TSP (m^2^)	Char Yield (wt.%)
EP-BZ-0	73 ± 3	1431 ± 33	68.6 ± 4.2	0.29 ± 0.02	21.4 ± 1.9	13.9 ± 1.8
EP-BZ-1	90 ± 4	310 ± 34	47.1 ± 3.7	0.12 ± 0.03	15.1 ± 1.1	35.3 ± 1.4
EP-BZ-2	95 ± 6	303 ± 21	44.1 ± 2.5	0.11 ± 0.03	12.2 ± 1.4	37.5 ± 3.2
EP-BZ-3	104 ± 6	221 ± 27	40.5 ± 3.2	0.09 ± 0.02	11.5 ± 1.3	42.6 ± 3.2

^a^ pk-HRR: Maximum value of heat release rate (HRR); ^b^ pk-SPR: Maximum value of smoke production rate (SPR).

## Data Availability

The original contributions presented in this study are included in the article. Further inquiries can be directed to the corresponding author.
